# Evolution of Plasticity in Response to Ethanol Between Recently Separated Populations of *D. melanogaster* With Different Ecological Histories

**DOI:** 10.1002/ece3.72874

**Published:** 2026-01-04

**Authors:** George Boateng‐Sarfo, Franz Scherping, Murad Mammadov, Sarah Signor

**Affiliations:** ^1^ Biological Sciences North Dakota State University Fargo North Dakota USA

## Abstract

While there is abundant theoretical work on the evolution of phenotype plasticity, empirical support has lagged. One model for the evolution of phenotype plasticity is by genetic accommodation. Under this model of evolution, when a population encounters a new environment there are widely variable responses among different genotypes, which are then pruned by selection into a single adaptive response. Because of the requirement to replicate genotypes, testing this prediction requires inbred lines as well as populations that are both adapted and not adapted to a resource. We previously demonstrated that *D. melanogaster* adapted to ethanol through genetic accommodation using *D. simulans* as an ancestral proxy lineage. However, we wondered how generalizable these results were. Here, we used a new population of *D. melanogaster* from France and an ancestral range population from Zambia and measured behavioral tolerance to ethanol exposure in multiple genotypes from each population, as well as genome‐wide gene expression and alternative splicing in response to ethanol using RNA sequencing. We found that the Zambian *D. melanogaster* have lower tolerance to ethanol than the French *D. melanogaster* , with the Zambian flies becoming sedated while the French flies remain active under the same exposure. At the transcriptional level, Zambian genotypes showed extensive genotype‐specific changes in gene expression and splicing in response to ethanol exposure, while the French genotypes showed relatively modest and fewer genotype‐specific changes, consistent with having a more uniform, population response. We also found that gene expression and splicing appear to evolve independently of one another and that the splicing response to ethanol is largely distinct between populations. Thus, we have independently replicated evidence for evolution by genetic accommodation in *D. melanogaster* , suggesting that the evolution of plasticity may be an important contributor to the ability to exploit novel resources.

## Graphical abstract

We identified: (1) differences in gene expression among each locale; (2) changes in gene expression in response to treatment; (3) genotype‐specific changes in gene expression in response to treatment.

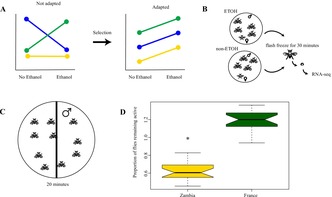

## 1 Introduction

**FIGURE 1:**
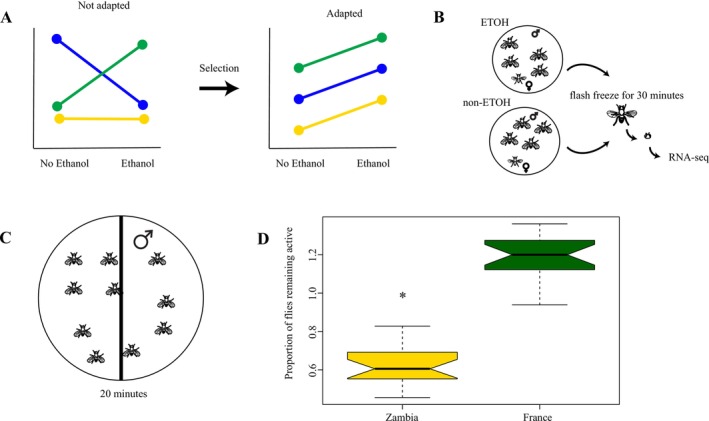
(A) An illustration of the predictions for phenotypic plasticity and the previously observed pattern in *D. melanogaster* and *D. simulans* . Each line represents a different genotype for a single trait, for example a gene whose expression changes in response to ethanol. If the species is not adapted to ethanol ( *D. simulans* ) you would expect to see that different genotypes respond differently, that is, genotype by environment interactions. This is what we found in *D. simulans* . If the species is adapted to ethanol, you would expect each genotype to respond the same way (right). This is what we found in cosmopolitan *D. melanogaster* from California. What we are testing here is whether this prediction holds true between cosmopolitan *D. melanogaster* (France) and those from the native range of *D. melanogaster* (Zambia) who are not adapted to ethanol. (B) An illustration of the environment that each *Drosophila* male was exposed to during the experiment. Each chamber contained 24 male flies and several females. The males were collected and flash frozen after 30 min. After flash freezing, their heads were isolated for RNA‐seq. This was done for three genotypes each of Zambian *D. melanogaster* and French *D. melanogaster* . (C) An example of the behavioral setup used to confirm that the two populations of *D. melanogaster* have different responses to ethanol. Every minute for 20 min, the number of flies who crossed the midline of the plate were recorded. The substrate contains 15% ethanol. (D) The proportion of flies that were not sedated and able to cross the midline of the plate was significantly higher in French populations of *D. melanogaster* .

The adaptive evolution of phenotype plasticity is theoretically predicted to occur through the process of genetic accommodation (Sun et al. 2019; Schlichting and Wund 2014; Jones and Robinson 2018; Lande 2014, 2015; Via and Lande 1985). Genetic accommodation proceeds through several stages, the first of which is a population encountering a new environment. Given that they are not adapted to that environment, there will be considerable variation between individuals in how they respond to the environment (Figure 1) (Schlichting and Wund 2014; West‐Eberhard 2005; Ghalambor et al. 2007; Robinson 2013; Morris et al. 2014; Signor and Nuzhdin 2019). At this stage, the set of plastic responses in the population has not been selected on; thus, they may be adaptive, deleterious, or neutral (Schlichting 2008; Gibson 2009; Hayden et al. 2011; Paaby and Rockman 2014). Adaptation to the novel environment will entail pruning of the plastic responses in the population to a single response that maximizes fitness (Baldwin 1896). However, it must also be adaptive to retain a plastic response rather than a fixed change (Via and Lande 1985; Guntrip and Sibly 1998; Lande 2009; Matzkin 2012; Huang et al. 2016). This process is theoretically sound but has little empirical support (Via and Lande 1985; Lande 2009; Chevin and Lande 2015).

One example where genetic accommodation was empirically demonstrated comes from *Drosophila*, where adaptation to ethanol in *D. simulans* and *D. melanogaster* met the expectations for genetic accommodation (Signor and Nuzhdin 2019). Cosmopolitan populations of *D. melanogaster* are tolerant of ethanol and are found feeding and ovipositing on resources with ethanol concentrations greater than 8% (McKenzie and McKechnie 1979; Gibson et al. 1981). Low tolerance to ethanol is the “ancestral state,” as most drosophilids are not tolerant to ethanol, and as such *D. simulans* avoids ethanol‐rich substrates (Merçot et al. 1994; Parsons and King 1977). Signor (Signor 2020) showed that four guidelines for establishing that the evolution of plasticity by genetic accommodation were met in this species pair (Levis and Pfennig 2016; Jones and Robinson 2018). First, the focal trait must be environmentally induced (ethanol) in a derived lineage (*D. melanogaster*, Winters, CA) and an ancestral‐proxy lineage (*D. simulans*, Zuma Beach, CA). Second, cryptic genetic variation must be uncovered when the ancestral proxy lineage is exposed to the derived environment—we found that in *D. simulans* each genotype interacted very differently to ethanol compared with *D. melanogaster* , where each genotype responded the same. This also relates to the third and fourth requirement, that the trait must show evidence of evolutionary change in the derived lineage and that the focal trait must exhibit evidence of adaptive refinement in the derived lineage. In summary, *D. simulans* exhibited extensive variation between genotypes in the plastic response to ethanol, evidence that cryptic genetic variation had not been removed by selection. Cosmopolitan *D. melanogaster* had a single plastic response to ethanol, evidence that selection has removed differences between genotypes.

Although this was strong evidence of evolution by genetic accommodation, and *D. simulans* does show the ancestral trait of low ethanol tolerance, we wondered how generalizable these results were and if we would find the same evidence if we compared a different population of cosmopolitan *D. melanogaster* (France) to African *D. melanogaster* (Zambia). African *D. melanogaster* in more remote locations (i.e., they do not show evidence of cosmopolitan admixture) still show the ancestral trait of low ethanol tolerance (Sprengelmeyer et al. 2019). Demographic models suggest that *D. melanogaster* began its expansion out of Africa 10,000 years ago and colonized Europe approximately 2000 years ago (Sprengelmeyer and Pool 2021; Sprengelmeyer 2021). Thus, this represents a recently evolved difference between these populations. If we found the same evidence—namely, genotype specific plasticity in African *D. melanogaster* —between these more recently separated populations, this would be additional solid evidence for evolution by genetic accommodation.

To demonstrate this, we compare an ancestral‐range population from Zambia, which retains low tolerance, to a derived cosmopolitan population from France, which has experienced extensive exposure to ethanol‐rich human environments. First, we measure behavioral tolerance to ethanol exposure in multiple genotypes from each population, predicting that the French genotype will exhibit higher tolerance to ethanol than the Zambian genotypes. We then used RNA sequencing to quantify gene and transcript level differential expression between ethanol‐exposed and non‐ethanol‐exposed *D. melanogaster* from the France and Zambia populations and focused our analyses on the interaction between genotype and ethanol treatment to test whether genotypes from the Zambian population show more transcriptional response than genotypes from the French population. We used three genotypes with three replicates from each location to estimate the contribution of genotype to the response to ethanol treatment. Finally, we investigated ethanol‐induced splicing in the same genotypes to determine whether splicing could be implicated in the regulation of this plasticity. If ethanol resistance evolved via genetic accommodation, we expect ancestral Zambian genotypes to show greater genotype‐specific plasticity, while the French population should show more uniform, typical population response. What we found was that this system supports our conclusion of evolution by genetic accommodation. Both populations have approximately the same response to treatment overall; however, in the Zambian population, each genotype responds differently to ethanol. In the French population, there is not a large contribution of genotype to the response to ethanol, suggesting that the plastic response has been pruned by selection. Furthermore, the genes that contribute to the response to treatment are consistent with our previous work on this system (Signor and Nuzhdin 2019). This is independent replication, using a more accurate ancestral proxy lineage, that fits the model of evolution by genetic accommodation. Replication of many studies is difficult, much less a system such as genetic accommodation which is quite difficult to demonstrate experimentally. Genetic accommodation has remained largely theoretical up until now, and we have demonstrated, with replication, its evolution in *Drosophila*. This is strong additional evidence for the importance of genetic accommodation in the evolution of plasticity.

## 2 Methods

### 2.1 Fly Strains

To compare the ethanol responses between the ancestral‐range and derived population of *D. melanogaster* , we used three wild derived genotypes from Zambia (ZI274N, ZI31N, ZI418N) and three from France (FR109, FR112N, FR113N). These fly strains were generously provided by John Pool (UW‐Madison) and originated either from France or Zambia (Lack et al. 2016a; Pool et al. 2012). The samples from Zambia were previously confirmed to be the most diverse among all the globally sampled strains, with minimal non‐African admixture, suggesting they come from the ancestral range of *D. melanogaster* (Pool et al. 2012; Lack et al. 2016b). The French lines originated from a cosmopolitan European population that has experienced long‐term association with human, ethanol‐rich environment (Lack et al. 2016a).

All six genotypes are wild‐derived laboratory stocks that have undergone inbreeding and are therefore substantially, though not perfectly homozygous. Using these inbreeds allowed us to minimize within‐genotype variation and thus increase our ability to detect genotype by ethanol interaction and consistent population level differences. We note that natural populations are outbred and thus typically are more heterozygous.

### 2.2 Fly Collection

To minimize variation due to non‐focal effects, all collection populations were set up with 10 one‐day‐old individuals of each sex from each genotype. The flies were reared on a standard Bloomington cornmeal medium at 25°C with a 12‐h light/12‐h dark cycle. After eight to nine days, the vials were cleared and males were collected for ethanol exposure assays and RNA‐seq. For each genotype, we collected 30 mated males within a three‐day window (3–5 days) and used these flies for both behavioral assays and transcriptomic profiling. Each of the three replicates per genotype (per treatment) was derived from an independent rearing, so that each vial is made up of a separate biological replicate and pseudo‐replication is avoided.

### 2.3 Experimental Setup

The flies were sedated through exposure to cold for 20 min and placed in petri dish lids with a paintbrush. Each petri dish contained 5 mL of standard grapefruit fly media or media in which 15% of the water had been replaced by ethanol. They were allowed to acclimate for 10 min prior to timing the 30‐min exposure (Figure 1A,B). This acclimation period is standard for behavioral assays, as it is long enough for the initial startle response to ethanol to have concluded; however, here it was included to standardize data with past observations (Jones and Robinson 2018; Lande 2014, 2015; Via and Lande 1985; West‐Eberhard 2005; Ghalambor et al. 2007). The assays were conducted within a 2 h window after dawn, the period in which the flies are most active (Robinson 2013; Morris et al. 2014; Signor and Nuzhdin 2019). Replicates were conducted randomly across days under standardized conditions (25°C, 70% humidity). At the conclusion of the assay, the flies were flash frozen in liquid nitrogen. Frozen nested sieves were used to separate their bodies from their heads, limbs, and wings. Heads were collected for sequencing.

### 2.4 Behavioral Assays

Differences in the response to ethanol have been previously observed in African and cosmopolitan *D. melanogaster* (Fry et al. 2008; Merçot et al. 1994; Fry et al. 2004; David and Kitagawa 1982). However, it was not demonstrated in the exact lines used here; therefore, we confirmed that African populations had a lower tolerance for ethanol. We performed three replicates of each of the six genotypes used in this study (Figure 1). A lower tolerance for ethanol would be indicated by a quicker progression through the euphoric stage of alcohol exposure to sedation (Via and Lande 1985; Baldwin 1896). The assays were conducted within a 2‐h window after dawn, to standardize for the effect of circadian rhythms. Replicates were conducted randomly under standardized conditions (25°C, 70% humidity). Approximately 30 male flies from a single genotype were sedated in a refrigerator for 10 min and then placed in a petri dish with 5 mL of grapefruit media in which 15% of the water had been replaced with ethanol (Figure 1B). The petri dish was bisected by a black line, and every minute the flies were observed for 10 s (Figure 1C). The number of flies that crossed the black line was recorded as a proxy for activity level, indicating that the flies were not sedated if they crossed the black line. This was done for 10 min. Please note that a behavioral analysis of this exposure to ethanol has been published and shows evidence of intoxication as well as genotype‐specific differences in the behavioral response to ethanol (Signor and Nuzhdin 2019; David and Kitagawa 1982; Signor et al. 2017a; Signor and Nuzhdin 2018; Signor et al. 2017b).

### 2.5 RNA Sequencing

To quantify population and genotype differences in gene expression responses to ethanol, RNA was extracted from the heads of 30 male flies using the NucleoZol one phase RNA purification kit (Macherey‐Nagel). For each population, we generated three biological replicates, each corresponding to an independent rearing vial (three replicates per population by genotypes by treatment) and this design was identical for both populations. Library preparation and sequencing were performed by BGI (Wuhan, China). The libraries were barcoded and pooled, and 2 million reads were generated per library on the illumina NextSeq. The data were demultiplexed prior to delivery.

### 2.6 Differential Expression Analysis

Adapters were trimmed and low‐quality reads in the raw fastq files were discarded using fastp (Chen et al. 2018). The abundances of the transcripts were quantified against the *D. melanogaster* reference transcriptome v.6.49 from Flybase using salmon v1.3.0 (Patro et al. 2017). The transcript level quantification was imported into R and summarized to gene‐level counts with tximport, using the *D. melanogaster* v6.49 GTF file and the GenomicFeatures R package (Soneson et al. 2016; Lawrence et al. 2013).

To determine how ethanol exposure alters gene expression within and between populations, and to test whether genotypes from the ancestral‐range population show more genotype‐specific transcriptional responses than genotypes from derived population, we performed differential expression analyses using DESeq2 (Love et al. 2014). This allowed us to model the read count for each gene using a negative binomial generalized linear model. Specifically, when we built our model for the DESeq2 step, we included terms for population (France vs. Zambia), genotype (the six inbred lines), ethanol treatment (ethanol treated samples vs. non‐ethanol treated samples) and the ethanol × genotype interaction. These terms allowed us to ask if ethanol exposure alone has any effect on gene expression, whether genotypes differ in their average expression and finally, if genotypes differ in the way they respond to ethanol (ethanol × genotype). Hence, we focus our differential gene expression analyses on the interaction term when discussing genotype‐specific transcriptional response to ethanol, unless otherwise stated.

For each term of interest, we obtain *p*‐values which were adjusted for multiple testing with Benjamini‐Hochberg correction (Benjamini and Hochberg 1995; Love et al. 2014) to control the false discovery rate. Genes with an adjusted p‐value ≤ 0.05 were considered differentially expressed. The results of the differential expression analysis were visualized to identify significant genes. However, for analyses of “core component of the response to ethanol,” we defined core expression genes as those that were significantly differentially expressed for the ethanol × genotype interaction term in both populations.

### 2.7 Alternative Splicing Analysis

To determine whether ethanol exposure induces population‐specific changes in alternative splicing, and whether splicing response parallels or is different from changes in overall gene expression, we quantified ethanol induced splicing events separately in the French and Zambian population.

We mapped the reads were mapped to the *D. melanogaster* v.6.49 genome with STAR aligner v.2.7.10a (Dobin et al. 2013). Then we quantify alternative splicing events using rMATs (turbo) (v. 4.1.2) (Shen et al. 2014; Park et al. 2013; Shen et al. 2012) and the *D. melanogaster* annotation file v. 6.49 from Flybase (Gramates et al. 2022). We did not allow unannotated splice sites and considered four classes of events, which are alternative 5′ splice sites (A5SS), skipped exons (SE), retained introns (RI), and alternative 3′ splice sites (A3SS) for downstream analyses. For each population, rMATS compared ethanol treated and non‐ethanol treated samples to identify events with significant changes in the percent spliced‐in (PSI) upon ethanol exposure. We limited our analysis to events with FDR <0.05 and ΔPSI > 0.2. In addition, splice detection analysis does not include interaction terms, therefore this analysis was limited to the effect of ethanol in each population. Then, we defined core splicing events as those that were significantly affected by ethanol in both populations and occurred in the same gene and event class. Finally, We collapsed the splicing events to gene level and compared those genes with the significantly differentially expressed genes to identify genes that are implicated in splicing. The scripts used for these analyses can be found here: https://github.com/gsarfo‐boateng/EvolutionofPlasticityinResponsetoEthanol.

## 3 Results

### 3.1 Behavioral Assays

To quantify differences in ethanol tolerance between ancestral‐range and derived population, we performed behavioral assays of acute ethanol exposure in multiple genotypes from each population. *D. melanogaster* from Zambia had a significantly lower tolerance to ethanol than French *D. melanogaster* (Figure 1C,D, two‐tailed *t*‐test *p* < 0.0001). The French *D. melanogaster* were able to stay in the euphoric stage of ethanol exposure without becoming sedated, while the Zambian *D. melanogaster* became sedated. This confirms the expected difference in ethanol tolerance between African and cosmopolitan *D. melanogaster* . As such, we know that cosmopolitan *D. melanogaster* are adapted to ethanol exposure and Zambian *D. melanogaster* are not.

### 3.2 Changes in Gene Expression

**FIGURE 2:**
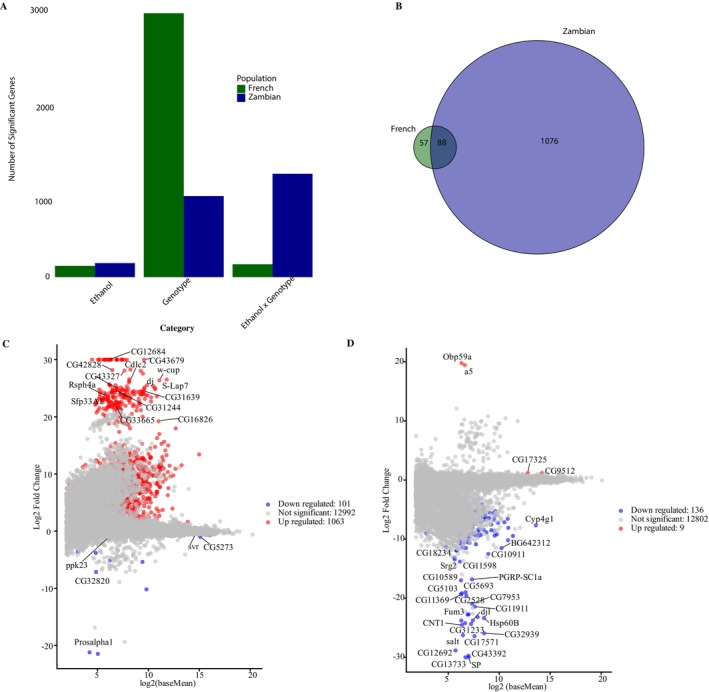
(A) The number of genes that significantly changed in relative abundance (adjusted *p*‐value ≤ 0.05) for each term in the differential expression model (ethanol treatment, genotype, and ethanol by genotype interaction) in *D. melanogaster* from the French and Zambian populations. (B) A Venn diagram showing the overlap between genes with a significant differential genes in the ethanol by genotype interaction (adjusted *p*‐value ≤ 0.05) between the French and Zambian populations. A total of 88 genes show significant ethanol by genotype interaction in both populations, with 57 genes unique to the French population and 1076 genes unique to the Zambian population. (C) Scatter plot of genes in the Zambian population, showing the estimated ethanol by genotype interaction effect (y‐axis; difference in ethanol‐induced expression change among genotypes) versus mean expression (*x*‐axis). Genes with a significant ethanol by genotype interaction (adjusted *p*‐value ≤ 0.05) are highlighted. (D) As in C, but for the French population.

Based on the ethanol‐treatment term in our DESeq2 model, we identified 158 significantly differentially genes in the Zambian population, compared with 127 genes in the French *D. melanogaster* (Figure 2; Data S1 and S2). However, the most striking difference is in the interaction term between genotype and treatment, where cosmopolitan *D. melanogaster* exhibited only 145 changes while Zambian *D. melanogaster* had 1164 genes that showed differential expression, an eight‐fold difference between populations (Figure 2). One explanation for the large effect of genotype in Zambian *D. melanogaster* compared with French *D. melanogaster* would be more variation attributable to genotype overall in Zambian *D. melanogaster* , particularly because the out of Africa expansion likely included a bottleneck (Li and Stephan 2006; Ometto et al. 2005; Haddrill et al. 2005; Li et al. 1999). However, this does not appear to be the case as in French *D. melanogaster* 2972 genes are differentially expressed between genotypes while in Zambian *D. melanogaster* only 913 genes differ between the genotypes. This implies that, if the Zambian samples had substantially lower variance within the genotypes, we would expect an increase in significance for both the genotype main effect and the ethanol by genotype interaction, rather than fewer genotype main effect genes in the Zambia and many more interaction genes. This contrast suggests that the number of significant genes in the interaction term in the Zambia reflects stronger genotype‐specific responses to ethanol treatment rather than a simple artifact of reduced variation among replicates.

In addition to the striking differences in the number of genes responding to ethanol by genotype in each population, the majority of genes in the Zambian population were upregulated (Figure 2C). A total of 1063 genes were up‐regulated in the Zambian population relative to the nine up‐regulated genes in the French population. Some genes were significant for more than one component of variance and they are double counted in those tallies, as they can have separate effects for different components of variance. The number of downregulated genes was consistent across populations, as there were 101 down‐regulated genes in the Zambian *D. melanogaster* and 136 in the French *D. melanogaster* (Figure 2C,D). This suggests that the adaptive response to ethanol may in part be the suppression of a response.

### 3.3 Core Components of the Response to Ethanol

Genes that show a significant ethanol by genotype interaction in both populations of *D. melanogaster* are likely to be core components of the genotype‐specific response to ethanol. In total, 136 genes had a response to ethanol in the Zambian and French populations of *D. melanogaster* (though not necessarily the same response), including *Drat, Pinocchio, cabut, sugarbabe*, and *Fatty Acid Synthase 2*. Compared to several other studies, four of the aforementioned genes are repeatedly implicated (*Drat, Pinocchio, cabut, sugarbabe*) (Morozova et al. 2006, 2011; Signor and Nuzhdin 2019; Kong et al. 2010). In addition, in at least two other studies and in our data set *FASN2, betaTub65B, AcCoAS, Pgd*, and *CG13607* were implicated in the response to ethanol (Morozova et al. 2006, 2011; Signor and Nuzhdin 2019; Kong et al. 2010). This suggests that these are core components of the response to ethanol, given that in many studies overlap between gene expression data sets can be low. Interestingly, the direction and magnitude of change of *Drat, Pinocchio, cabut*, and *sugarbabe* was the same between populations, suggesting they may be part of a conserved response to ethanol rather than part of the adaptive response in the French population.

### 3.4 Alternative Splicing

**FIGURE 3:**
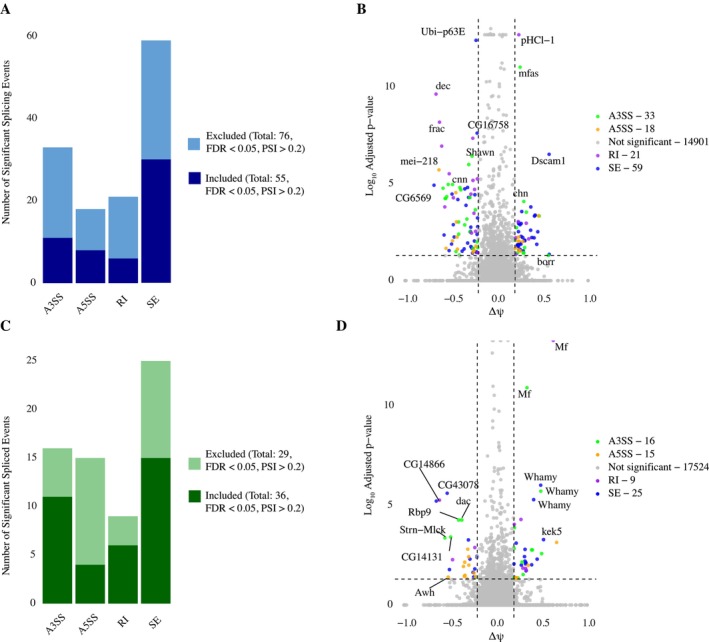
(A) Bar plot that shows the number of significant splicing events in the Zambian population after ethanol exposure. (B) The volcano plot illustrates genes with inclusion and exclusion level (PSI) difference (*x*‐axis) in the French and Zambian population corresponding to the False Discovery Rate (FDR) (*y*‐axis). Each data point represents a splicing event obtained from rMARTs. Events with ΔPSI > 0 (< 0) indicate exon inclusion in the Zambian population relative to the control samples. Gray denotes splicing events that did not reach significance (adjusted *p* > 0.05 or −0.2 < ΔPSI < 0.2). Four splicing event types (A3SS, A5SS, RI, ES) are indicated by different colors (green, orange, purple, and blue, respectively). (C) Bar plot that shows the number of significant splicing events in the French population after ethanol exposure. (D) The volcano plot shows genes with inclusion and exclusion level (PSI) difference (*x* axis) in the French population.

**FIGURE 4:**
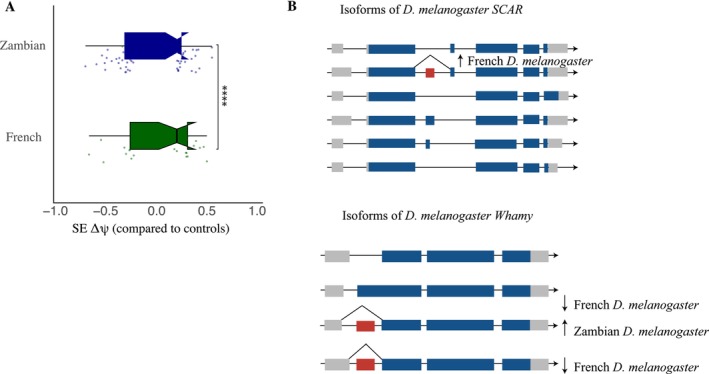
(A) A plot box illustrating the difference in the number of skipped exons in between the French and Zambian population. This significant difference is supported by a fisher's test = 0.000279. (B) Two examples of the complex splicing exon skipping events in *Whamy* and *SCAR*.

Next we asked whether ethanol exposure also alters alternative splicing, and whether these splicing responses differ between the ancestral range and the derived populations. In general, the splicing response to ethanol was larger in Zambian populations of *D. melanogaster* , with 88 Skipped Exon (SE) (54 France), 57 Alternative 3′ Spliced Site (A3′SS) (32 France), 43 Intron Retention (RI) (25 France), and 41 Alternative 5′ Splice Sites (A5SS) (31 France) (Figure 3A–D, Data S3 and S4). Across both groups, SE was the most common splicing event, followed by A3′SS usage. This prevalence of SE events was supported by a Fisher's exact test (*p* = 0.000279; Figure 4A), reinforcing the dominant role of exon skipping in the ethanol‐induced splicing response. Thiryt‐four genes and 32 events are shared between populations among all these categories, but of those, only five are in the same direction, suggesting that the response to ethanol is quite distinct between populations. Interestingly, several Zambian genes previously associated with neural function—including *slowpoke*, *CASK*, and *dunce—were* found to undergo ethanol‐induced splicing changes. Of these, only *slowpoke* has been previously documented to exhibit alternative splicing in response to ethanol exposure (Cowmeadow et al. 2006, 2005; Rodan and Rothenfluh 2010). In the adapted French population, a number of genes known to interact with one another in muscle specification are implicated, including *spalt major, Myofilin, Stretchin‐Mlck, frayed, myospheroid, SCAR, sallimus, tropomodulin, upheld*, and *wings upA* (Figure 3) (Skoulakis Crittenden et al. 2018; Lin et al. 1996; Sivachenko et al. 2013). Interestingly, *mef2* is thought to be a general regulator of ethanol sedation and is responsible for both muscle development and regulation of gene expression in neural tissue (Schmitt et al. 2019; Talikoti 2021; Adhikari et al. 2018). Indeed, *mef2* has a splicing difference in the Zambian population, and 39 genes in the Zambian population were also uncovered in a screen of *mef2* targets, including *Myosin heavy chain, shaking B*, and *dunce* (Talikoti 2021). In the French population, 33 genes were also implicated in this screen of *mef2* targets, including *fruitless, dachshund, Bruce, myospheroid*, and *frayed. Mef2* and *spalt major* are both myogenic regulators, but their relationship to each other is not clear. This raises the interesting possibility that the myogenic pathways may be an important part of the splicing response to ethanol. Overall, overlap with previous work on alternative splicing in response to ethanol is low, with *Slowpoke binding protein, Syncrip, tweek, wings up A, midline fasciclin, Myosin heavy chain*, and *Rab3 interacting molecule* being found in at least one population here and previous work (Signor and Nuzhdin 2018, 2019; Petruccelli et al. 2018).

### 3.5 Core Components of the Splicing Response

Splicing changes that occur in the same gene are likely part of the core response to ethanol, even when the direction of exon change inclusion differs between populations. In the Zambian data set, nine genes showed overlap between differential expression and alternative splicing. In contrast, we did not observe such overlap in the French flies. In the Zambian population overlapping genes include known candidates such as *Syncrip* and *NFAT*, as well as new candidates such as *smooth, myospheroid*, and *suppressor of hairy wing*. *Myospheroid, NFAT, smooth, scalloped*, and *terribly reduced optic lobes* altered splicing in the same direction, suggesting they are not part of the adaptive response to ethanol. However, this is complicated; for example, a skipped exon in *smooth* changed in opposite directions in the two populations, while 3′ alternative splicing changed in the same direction, suggesting there are likely still differences in isoform abundance at this gene (see Figure 4B for examples of complicated splicing responses). *Syncrip* was found in both populations changing in the opposite direction and was implicated in previous work in alternative splicing in response to ethanol (Signor and Nuzhdin 2018, 2019).

### 3.6 Splicing and Gene Expression Evolution Are Distinct

For the response to ethanol, the French population did not share any genes between the gene expression and splicing analysis. Furthermore, for the differential gene expression analysis of genotype by ethanol and the splicing response to ethanol, no genes were shared in the French population. In the Zambian population, the number of genes shared between the differentially expressed genes for the interaction term and the splicing response to ethanol was nine (out of more than a thousand that changed in the Zambian expression differences). Thus, the splicing and gene expression differences in response to ethanol are largely distinct.

## 4 Discussion

There are very few examples where the evolution of genetic accommodation has been demonstrated, in large part due to the difficulty of estimating genotype by environment interactions with replicated genotypes. Evolution by genetic accommodation has remained largely theoretical. Here we have demonstrated, using new populations of *D. melanogaster* , that the evolution of ethanol tolerance in *D. melanogaster* has occurred through genetic accommodation. This replicates previous results using *D. simulans* as the ancestral proxy lineage and a different population of *D. melanogaster* (Signor and Nuzhdin 2019). In *D. simulans,* there was abundant variation in how each genotype responds to ethanol, and in cosmopolitan *D. melanogaster* there was little variation (Figure 1A). However, it is possible that the observed pattern was isolated to this species pair and not a generalizable pattern. Furthermore, while *D. simulans* is the sister species to *D. melanogaster* and is not adapted to ethanol, populations of *D. melanogaster* with the ancestral state are more directly comparable. Here, we have demonstrated that genetic accommodation has occurred within the *D. melanogaster* lineage in response to ethanol. Ancestral range *D. melanogaster* that are not adapted to ethanol show the same pattern of genotype‐specific responses as *D. simulans* . Without selection on the response to ethanol, environmentally induced variants can accumulate as cryptic genetic variation and manifest as greater variation between genotypes (Schlichting and Wund 2014; Rutherford 2000; Gibson and Dworkin 2004; Hermisson and Wagner 2004). Because ethanol is a patchy resource the response to ethanol is expected to be selected as an optimal plastic response (Via and Lande 1985; Guntrip and Sibly 1998; Lande 2009; Matzkin 2012; Huang et al. 2016). Given that replication of gene expression studies can often be limited, this is strong evidence for evolution by genetic accommodation. This provides one of the only sure cases where evolution by genetic accommodation has been demonstrated biologically.

Ancestral range and cosmopolitan *D. melanogaster* fit the criteria for establishing evolution by genetic accommodation (Levis and Pfennig 2016; Jones and Robinson 2018). Using ancestral range *D. melanogaster* as a proxy lineage for ancestral *D. melanogaster* , the focal trait can be environmentally induced by exposing them to ethanol. This exposure uncovers cryptic genetic variation—a large increase in genotype‐specific responses to ethanol. Furthermore, while our previous work showed a loss of genotype‐specific response in Californian cosmopolitan *D. melanogaster* , this work confirmed this loss of genotype‐specific responses in a second French population of *D. melanogaster* (Signor and Nuzhdin 2019). Lastly, the focal trait shows evidence of adaptive refinement in cosmopolitan *D. melanogaster* —confirmed here with the increased resistance of French *D. melanogaster* and recorded elsewhere in terms of preferential oviposition in ethanol‐rich substrate (Milan et al. 2012; Pohl et al. 2012).

We also demonstrate here that gene expression and splicing have evolved independently, confirming that they have a separate genetic basis. This is consistent with recent predictions that suggest that separate responses for expression and splicing will be common and an important contributor to plasticity over short timescales (Verta and Jacobs 2022). The implication is that splicing and gene expression will also have distinct functions and affect different pathways, and indeed the two data sets contain genes with very different functions—for example, the French splicing data set contains many *mef2* interacting genes and genes thought to be involved in muscle/brain specification, while the gene expression data sets do not.

Previously we reported that in *D. simulans* the response to ethanol was enriched for non‐protein coding genes as well as nested genes (genes in the introns of other genes). We hypothesized that cryptic genetic variation affecting gene expression preferentially accumulates in intronic non‐protein coding genes due to lower selective constraint on expression. However, we did not find any enrichment in African *D. melanogaster* suggesting this is not a general feature of the accumulation of cryptic genetic variation. We also did not find a larger contribution of splicing in the adapted population, though we did find a larger contribution of splicing in California *D. melanogaster* compared with *D. simulans* . However, the method used to quantify splicing is very different between these manuscripts, as the previous paper used a more bespoke pipeline (Signor and Nuzhdin 2019; Sprengelmeyer and Pool 2021).

In our analysis of gene expression differences, we uncovered some of the same genes which have been implicated in other studies including our own—*Drat*, *cabut*, *sugarbabe*, and *Pinocchio*, to name just a few. *cabut* and *Pinocchio* are also thought to be targets of *mef2*, potentially one of the core regulators of the response to ethanol (Schmitt et al. 2019; Talikoti 2021). While gene expression differences did not overlap considerably with the list of *mef2* targets, in the French population alternative splicing did, and contained many other myogenic genes that either interact with *spalt major* or have been implicated in that pathway. While *mef2* and *spalt major* are referred to as myogenic genes, *mef2* is required for mushroom body development, neuronal plasticity, and circadian rhythms (Schmitt et al. 2019; Talikoti 2021; Skoulakis Crittenden et al. 2018; Adhikari et al. 2018; Lin et al. 1996; Sivachenko et al. 2013). *Spalt major* has a role in the brain and is a known target of *terribly reduced optic lobes* which was also implicated in splicing differences in the French population. Many of these genes appear to be transcription factors that may play a role in many essential functions, interacting with the same set of genes but deployed in different contexts. For example, *spalt major* is responsible for alternative splicing of *Myofilin* in developing muscles, and we see changes in the splicing of both in this data set, but likely they are performing a neural function (Spletter et al. 2015).

The evolution of phenotype plasticity is not well understood, with few examples where genotype can be included as a factor to understand variation in the plastic response within a population. The patterns observed in Zambian *D. melanogaster* suggest that abundant genotype by environment interactions have accumulated neutrally and become uncovered in response to a novel environment. In contrast, in French *D. melanogaster* the ethanol environment is not novel and variation in plasticity has been selected out in favor of an adaptive phenotypic response. This represents an independent confirmation of genetic accommodation in *D. melanogaster* , using a more appropriate ancestral proxy lineage to understand the dynamics of ethanol adaptation. This study supports the evolution of ethanol tolerance through genetic accommodation in *D. melanogaster* , confirming theoretical predictions about how phenotype plasticity evolves.

## Author Contributions

**George Boateng‐Sarfo:** data curation (equal), formal analysis (equal), investigation (equal), methodology (equal), validation (equal), visualization (equal), writing – original draft (equal), writing – review and editing (equal). **Franz Scherping:** data curation (equal). **Murad Mammadov:** data curation (equal). **Sarah Signor:** conceptualization (equal), data curation (equal), formal analysis (equal), funding acquisition (equal), investigation (equal), methodology (equal), project administration (equal), supervision (equal), validation (equal), visualization (equal), writing – original draft (equal), writing – review and editing (equal).

## Funding

This work was supported by the National Science Foundation Established Program to Stimulate Competitive Research (NSF‐EPSCoR‐1826834 and NSF‐EPSCoR‐2032756) to SS.

## Conflicts of Interest

The authors declare no conflicts of interest.

## Supporting information


**Data S1:** The DESeq2 results for the Zambian population. The Excel file has three sheets: (1) the ethanol treatment effect (ethanol‐treated vs control across genotypes), (2) the genotype Ã—treatment (interaction) term, and (3) the comparison between genotypes (genotype main effect).

**Data S2:** The DESeq2 results for the French population. The Excel file has the same three sheets: (1) the ethanol treatment effect, (2) the genotype Ã—treatment (interaction) term, and (3) the comparison between genotypes.

**Data S3:** The alternative splicing results for the Zambian population. These results are based on comparisons of ethanol‐treated samples to controls. The file reports significant events for four splicing categories: retained intron (RI), alternative 3′ splice site (A3SS), alternative 5′ splice site (A5SS), and skipped exon (SE).

**Data S4:** The alternative splicing results for the French population. As in Data S3, the results are based on comparisons of ethanol‐treated samples to controls, and include the four splicing categories: retained intron (RI), alternative 3′ splice site (A3SS), alternative 5′ splice site (A5SS), and skipped exon (SE).

## Data Availability

All raw fastq files have been deposited on the National Center for Biotechnology Information under the Bioproject number PRJNA1347749. A gitHub link to the scripts used for the analysis has been provided [https://github.com/gsarfo_boateng/Evolution_of_Plasticity_in_Response_to_Ethanol].

## References

[ece372874-bib-0001] Adhikari, P. , D. Orozco , H. Randhawa , and F. W. Wolf . 2018. “Mef2 Induction of the Immediate Early Gene hr38/nr4a Is Terminated by sirt1 to Promote Ethanol Tolerance.” Genes, Brain, and Behavior 18: e12486. 10.1111/gbb.12486. 29726098 PMC6215524

[ece372874-bib-0002] Baldwin, J. M. 1896. “A New Factor in Evolution.” American Naturalist 30: 441–451.

[ece372874-bib-0003] Benjamini, Y. , and Y. Hochberg . 1995. “Controlling the False Discovery Rate: A Practical and Powerful Approach to Multiple Testing.” Journal of the Royal Statistical Society, Series B 57, no. 1: 289–300. 10.2307/2346101.

[ece372874-bib-0004] Chen, S. , Y. Zhou , Y. Chen , and J. Gu . 2018. “Fastp: An Ultra‐Fast All‐In‐One Fastq Preprocessor.” Biorxiv 34, no. 17: i884–i890. 10.1101/274100. PMC6129281 30423086

[ece372874-bib-0005] Chevin, L.‐M. , and R. Lande . 2015. “Evolution of Environmental Cues for Phenotypic Plasticity.” Evolution 69: 2767–2775. 10.1111/evo.12755. 26292649

[ece372874-bib-0006] Cowmeadow, R. B. , H. R. Krishnan , and N. S. Atkinson . 2005. “The Slowpoke Gene Is Necessary for Rapid Ethanol Tolerance in Drosophila.” Alcoholism: Clinical and Experimental Research 29: 1777–1786. 10.1097/01.alc.0000183232.56788.62. 16269907

[ece372874-bib-0007] Cowmeadow, R. B. , H. R. Krishnan , A. Ghezzi , Y. M. Al'Hasan , Y. Z. Wang , and N. S. Atkinson . 2006. “Ethanol Tolerance Caused by Slowpoke Induction in Drosophila.” Alcoholism, Clinical and Experimental Research 30: 745–753. 10.1111/j.1530-0277.2006.00087.x. 16634842

[ece372874-bib-0008] David, J. R. , and O. Kitagawa . 1982. “Possible Similarities in Ethanol Tolerance and Latitudinal Variations Between Drosophila Virilis and D. *Melanogaster* .” Japanese Journal of Genetics 57: 89–95. 10.1266/jjg.57.89.

[ece372874-bib-0009] Dobin, A. , C. A. Davis , F. Schlesinger , J. Drenkow , C. Zaleski , and S. Jha . 2013. “Star: Ultrafast Universal RNA‐Seq Aligner.” Bioinformatics 29: 15–21. 10.1093/bioinformatics/bts635. 23104886 PMC3530905

[ece372874-bib-0010] Fry, J. D. , C. M. Bahnck , M. Mikucki , N. Phadnis , and W. C. Slattery . 2004. “Dietary Ethanol Mediates Selection on Aldehyde Dehydrogenase Activity in *drosophila melanogaster* .” Integrative and Comparative Biology 44: 275–283. 10.1093/icb/44.4.275. 21676710

[ece372874-bib-0011] Fry, J. D. , K. Donlon , and M. Saweikis . 2008. “A Worldwide Polymorphism in Aldehyde Dehydrogenase in *Drosophila melanogaster* : Evidence for Selection Mediated by Dietary Ethanol.” Evolution 62: 66–75. 10.1111/j.1558-5646.2007.00288.x. 18070084

[ece372874-bib-0012] Ghalambor, C. K. , J. K. McKAY , S. P. Carroll , and D. N. Reznick . 2007. “Adaptive Versus Non‐Adaptive Phenotypic Plasticity and the Potential for Contemporary Adaptation in New Environments.” Functional Ecology 21: 394–407. 10.1111/j.1365-2435.2007.01283.x.

[ece372874-bib-0013] Gibson, G. 2009. “Decanalization and the Origin of Complex Disease.” Nature Reviews Genetics 10: 134–140. 10.1038/nrg2502. 19119265

[ece372874-bib-0014] Gibson, G. , and I. Dworkin . 2004. “Uncovering Cryptic Genetic Variation.” Nature Reviews Genetics 5: 681–690. 10.1038/nrg1426. 15372091

[ece372874-bib-0015] Gibson, J. , T. May , and A. Wilks . 1981. “Genetic Variation at the Alcohol Dehydrogenase Locus in *Drosophila melanogaster* in Relation to Environmental Variation: Ethanol Levels in Breeding Sites and Allozyme Frequencies.” Oecologia 51: 191–198. 10.1007/bf00540600. 28310081

[ece372874-bib-0016] Gramates, L. S. , J. Agapite , H. Attrill , et al. 2022. “Flybase: A Guided Tour of Highlighted Features.” Genetics 220: iyac035. 10.1093/genetics/iyac035. 35266522 PMC8982030

[ece372874-bib-0017] Guntrip, J. , and R. M. Sibly . 1998. “Phenotypic Plasticity, Genotype‐By‐Environment Interaction and the Analysis of Generalism and Specialization in *Callosobruchus maculatus* .” Heredity 81: 198–204. 10.1046/j.1365-2540.1998.00354.x.

[ece372874-bib-0018] Haddrill, P. R. , K. R. Thornton , B. Charlesworth , and P. Andolfatto . 2005. “Multilocus Patterns of Nucleotide Variability and the Demographic and Selection History of *Drosophila melanogaster* Populations.” Genome Research 15: 790–799. 10.1101/gr.3541005. 15930491 PMC1142469

[ece372874-bib-0019] Hayden, E. J. , E. Ferrada , and A. Wagner . 2011. “Cryptic Genetic Variation Promotes Rapid Evolutionary Adaptation in an RNA Enzyme.” Nature 474: 92–95. 10.1038/nature10083. 21637259

[ece372874-bib-0020] Hermisson, J. , and G. P. Wagner . 2004. “The Population Genetic Theory of Hidden Variation and Genetic Robustness.” Genetics 168: 2271–2284. 10.1534/genetics.104.029173. 15611191 PMC1448756

[ece372874-bib-0021] Huang, Y. , I. Tran , and A. F. Agrawal . 2016. “Does Genetic Variation Maintained by Environmental Heterogeneity Facilitate Adaptation to Novel Selection?” American 188: 27–37. 10.5061/dryad.kb830. 27322119

[ece372874-bib-0022] Jones, B. M. , and G. E. Robinson . 2018. “Genetic Accommodation and the Role of Ancestral Plasticity in the Evolution of Insect Eusociality.” Journal of Experimental Biology 221: jeb153163. 10.1242/jeb.153163. 30478152 PMC6288071

[ece372874-bib-0023] Kong, E. C. , L. Allouche , P. A. Chapot , et al. 2010. “Ethanol‐Regulated Genes That Contribute to Ethanol Sensitivity and Rapid Tolerance in Drosophila.” Alcoholism: Clinical and Experimental Research 34: 302–316. 10.1111/j.1530-0277.2009.01093.x. 19951294 PMC2903447

[ece372874-bib-0024] Lack, J. B. , J. D. Lange , A. B. Tang , R. B. Corbett‐Detig , and J. E. Pool . 2016a. “A Thousand Fly Genomes: An Expanded Drosophila Genome Nexus.” Preprint, bioRxiv. 10.1101/063537. PMC5100052 27687565

[ece372874-bib-0025] Lack, J. B. , J. D. Lange , A. D. Tang , R. B. Corbett‐Detig , and J. E. Pool . 2016b. “A Thousand Fly Genomes: An Expanded Drosophila Genome Nexus.” Molecular Biology and Evolution 33, no. 12: 3308–3313. 10.1093/molbev/msw195. 27687565 PMC5100052

[ece372874-bib-0026] Lande, R. 2009. “Adaptation to an Extraordinary Environment by Evolution of Phenotypic Plasticity and Genetic Assimilation.” Journal of Evolutionary Biology 22: 1435–1446. 10.1111/j.1420-9101.2009.01754.x. 19467134

[ece372874-bib-0027] Lande, R. 2014. “Evolution of Phenotypic Plasticity and Environmental Tolerance of a Labile Quantitative Character in a Fluctuating Environment.” Journal of Evolutionary Biology 27: 866–875. 10.1111/jeb.12360. 24724972

[ece372874-bib-0028] Lande, R. 2015. “Evolution of Phenotypic Plasticity in Colonizing Species.” Molecular Ecology 24: 2038–2045. 10.1111/mec.13037. 25558898

[ece372874-bib-0029] Lawrence, M. , W. Huber , H. Pagès , et al. 2013. “Software for Computing and Annotating Genomic Ranges.” PLoS Computational Biology 9: e1003118. 10.1371/journal.pcbi.1003118. 23950696 PMC3738458

[ece372874-bib-0030] Levis, N. A. , and D. W. Pfennig . 2016. “Evaluating ‘Plasticity‐First’ Evolution in Nature: Key Criteria and Empirical Approaches.” Trends in Ecology & Evolution 31: 563–574. 10.1016/j.tree.2016.03.012. 27067134

[ece372874-bib-0031] Li, H. , and W. Stephan . 2006. “Inferring the Demographic History and Rate of Adaptive Substitution in Drosophila.” PLoS Genetics 2: e166. 10.1371/journal.pgen.0020166. 17040129 PMC1599771

[ece372874-bib-0032] Li, Y. J. , Y. Satta , and N. Takahata . 1999. “Paleo‐Demography of the *drosophila melanogaster* Subgroup: Application of the Maximum Likelihood Method.” Genes & Genetic Systems 74: 117–127. 10650839 10.1266/ggs.74.117

[ece372874-bib-0033] Lin, X. , S. Shah , and R. F. Bulleit . 1996. “The Expression of mef2 Genes Is Implicated in Cns Neuronal Differentiation.” Molecular Brain Research 42: 307–316. 10.1016/s0169-328x(96)00135-0. 9013788

[ece372874-bib-0034] Love, M. I. , W. Huber , and S. Anders . 2014. “Moderated Estimation of Fold Change and Dispersion for RNA‐Seq Data With deseq2.” Genome Biology 15: 550. 10.1186/s13059-014-0550-8. 25516281 PMC4302049

[ece372874-bib-0035] Matzkin, L. M. 2012. “Population Transcriptomics of Cactus Host Shifts in *Drosophila mojavensis* .” Molecular Ecology 21: 2428–2439. 10.1111/j.1365-294x.2012.05549.x. 22512269

[ece372874-bib-0036] McKenzie, J. A. , and S. W. McKechnie . 1979. “A Comparative Study of Resource Utilization in Natural Populations of *Drosophila melanogaster* and *D. simulans* .” Oecologia 40: 299–309. 10.1007/bf00345326. 28309613

[ece372874-bib-0037] Merçot, H. , D. Defaye , P. Capy , E. Pla , and J. R. David . 1994. “Alcohol Tolerance, Adh Activity, and Ecological Niche of Drosophila Species.” Evolution 48, no. 3: 746–757. 28568255 10.1111/j.1558-5646.1994.tb01358.x PMC7163518

[ece372874-bib-0038] Milan, N. F. , B. Z. Kacsoh , and T. A. Schlenke . 2012. “Alcohol Consumption as Self‐Medication Against Blood‐Borne Parasites in the Fruit Fly.” Current Biology 22: 488–493. 10.1016/j.cub.2012.01.045. 22342747 PMC3311762

[ece372874-bib-0039] Morozova, T. , R. Anholt , and T. F. Mackay . 2006. “Transcriptional Response to Alcohol Exposure in *Drosophila melanogaster* .” Genome Biology 7: R95. 10.1186/gb-2006-7-10-r95. 17054780 PMC1794562

[ece372874-bib-0040] Morozova, T. V. , T. F. C. Mackay , and R. R. H. Anholt . 2011. “Transcriptional Networks for Alcohol Sensitivity in *Drosophila melanogaster* .” Genetics 187: 1193–1205. 10.1534/genetics.110.125229. 21270389 PMC3070527

[ece372874-bib-0041] Morris, M. R. J. , R. Richard , E. H. Leder , R. D. H. Barrett , N. Aubin‐Horth , and S. M. Rogers . 2014. “Gene Expression Plasticity Evolves in Response to Colonization of Freshwater Lakes in Threespine Stickleback.” Molecular Ecology 23: 3226–3240. 10.1111/mec.12820. 24889067

[ece372874-bib-0042] Ometto, L. , S. Glinka , D. D. Lorenzo , and W. Stephan . 2005. “Inferring the Effects of Demography and Selection on *Drosophila melanogaster* Populations From a Chromosome‐Wide Scan of DNA Variation.” Molecular Biology and Evolution 22: 2119–2130. 10.1093/molbev/msi207. 15987874

[ece372874-bib-0043] Paaby, A. B. , and M. V. Rockman . 2014. “Cryptic Genetic Variation: Evolution's Hidden Substrate.” Nature Reviews Genetics 15: 247–258. 10.1038/nrg3688. PMC4737706 24614309

[ece372874-bib-0044] Park, J. W. , C. Tokheim , S. Shen , and Y. Xing . 2013. “Identifying Differential Alternative Splicing Events From RNA Sequencing Data Using RNAseq‐Mats.” Methods in Molecular Biology and Evolution 1038: 171–179. 10.1007/978-1-62703-514-9_10 23872975

[ece372874-bib-0045] Parsons, P. A. , and S. B. King . 1977. “Ethanol: Larval Discrimination Between Two Drosophila Sibling Species.” Experientia 33: 898–899. 10.1007/bf01951269. 408175

[ece372874-bib-0046] Patro, R. , G. Duggal , M. I. Love , R. A. Irizarry , and C. Kingsford . 2017. “Salmon Provides Fast and Bias‐Aware Quantification of Transcript Expression.” Nature Methods 14: 417–419. 10.1038/nmeth.4197. 28263959 PMC5600148

[ece372874-bib-0047] Petruccelli, E. , M. Feyder , N. Ledru , Y. Jaques , E. Anderson , and K. R. Kaun . 2018. “Alcohol Activates Scabrous‐Notch to Influence Associated Memories.” Neuron 100: 1209–1223.e4. 10.1016/j.neuron.2018.10.005. 30482693 PMC6323638

[ece372874-bib-0048] Pohl, J. B. , B. A. Baldwin , B. L. Dinh , et al. 2012. “Ethanol Preference in *Drosophila melanogaster* Is Driven by Its Caloric Value.” Alcoholism, Clinical and Experimental Research 36: 1903–1912. 10.1111/j.1530-0277.2012.01817.x. 22551215 PMC3414655

[ece372874-bib-0049] Pool, J. E. , R. B. Corbett‐Detig , R. P. Sugino , and K. A. Stevens . 2012. “Population Genomics of Sub‐Saharan *Drosophila melanogaster* : African Diversity and Non‐African Admixture.” PLoS Genetics 8: e1003080. 10.1371/journal.pgen.1003080. 23284287 PMC3527209

[ece372874-bib-0050] Robinson, B. W. 2013. “Evolution of Growth by Genetic Accommodation in Icelandic Freshwater Stickleback.” Proceedings of the Royal Society B 280: 20132197. 10.1098/rspb.2013.2197. 24132309 PMC3813338

[ece372874-bib-0051] Rodan, A. R. , and A. Rothenfluh . 2010. “The Genetics of Behavioral Alcohol Responses in Drosophila.” International Review of Neurobiology 91: 25–51. 20813239 10.1016/S0074-7742(10)91002-7 PMC3531558

[ece372874-bib-0052] Rutherford, S. L. 2000. “From Genotype to Phenotype: Buffering Mechanisms and the Storage of Genetic Information.” BioEssays 22: 1095–1105. 10.1002/1521-1878(200012)22:12. 11084625

[ece372874-bib-0053] Schlichting, C. D. 2008. “Cryptic Genetic Variation, and Evolvability.” Annals of the New York Academy of Sciences 1133: 187–203. 10.1196/annals.1438.010. 18559822

[ece372874-bib-0054] Schlichting, C. D. , and M. A. Wund . 2014. “Phenotypic Plasticity and Epigenetic Marking: An Assessment of Evidence for Genetic Accommodation.” Evolution 68: 656–672. 10.1111/evo.12348. 24410266

[ece372874-bib-0055] Schmitt, R. E. , B. C. Shell , K. M. Lee , et al. 2019. “Convergent Evidence From Humans and *Drosophila melanogaster* Implicates the Transcription Factor mef2b/mef2 in Alcohol Sensitivity.” Alcoholism, Clinical and Experimental Research 43: 1872–1886. 10.1111/acer.14138. 31241765 PMC6721962

[ece372874-bib-0056] Shen, S. , J. W. Park , J. Huang , et al. 2012. “Mats: A Bayesian Framework for Flexible Detection of Differential Alternative Splicing From RNA‐Seq Data.” Nucleic Acids Research 40: e61. 10.1093/nar/gkr1291. 22266656 PMC3333886

[ece372874-bib-0057] Shen, S. , J. W. Park , Z. Lu , et al. 2014. “Rmats: Robust and Flexible Detection of Differential Alternative Splicing From Replicate RNA‐Seq Data.” Proceedings of the National Academy of Sciences 111: E5593–E5601. 10.1073/pnas.1419161111. PMC4280593 25480548

[ece372874-bib-0074] Signor, S. A. 2020. “Evolution of Plasticity in Response to Ethanol Between Sister Species With Different Ecological Histories (*Drosophila melanogaster* and *D. simulans*).” American Naturalist 196, no. 5: 620–633. 10.1086/711169. 33064591

[ece372874-bib-0058] Signor, S. A. , M. Abbasi , P. Marjoram , and S. V. Nuzhdin . 2017a. “Conservation of Social Effects (ψ) Between Two Species of Drosophila Despite Reversal of Sexual Dimorphism.” Ecology and Evolution 7: 10031–10041. 10.1002/ece3.3523. 29238534 PMC5723616

[ece372874-bib-0059] Signor, S. A. , M. Abbasi , P. Marjoram , and S. V. Nuzhdin . 2017b. “Social Effects for Locomotion Vary Between Environments in *Drosophila melanogaster* Females.” Evolution 71: 1765–1775. 10.1111/evo.13266. 28489252

[ece372874-bib-0061] Signor, S. , and S. Nuzhdin . 2018. “Dynamic Changes in Gene Expression and Alternative Splicing Mediate the Response to Acute Alcohol Exposure in *drosophila melanogaster* .” Heredity 121, no. 4: 342–360. 30143789 10.1038/s41437-018-0136-4 PMC6133934

[ece372874-bib-0060] Signor, S. A. , and S. V. Nuzhdin . 2019. “Evolution of Phenotypic Plasticity in Response to Ethanol Between Sister Species With Different Ecological Histories (*Drosophila melanogaster* and *D. simulans*).” Preprint, bioRxiv. 10.1101/386334. 33064591

[ece372874-bib-0062] Sivachenko, A. , Y. Li , K. C. Abruzzi , and M. Rosbash . 2013. “The Transcription Factor mef2 Links the Drosophila Core Clock to fas2.” Neuronal Morphology, and Circadian Behavior 79: 281–292. 10.1016/j.neuron.2013.05.015. PMC3859024 23889933

[ece372874-bib-0063] Skoulakis Crittenden, J. R. , E. M. C. Goldstein , and E. S. Davis . 2018. “Drosophila mef2 Is Essential for Normal Mushroom Body and Wing Development.” Biology Open 7: bio035618. 10.1242/bio.035618. 30115617 PMC6176937

[ece372874-bib-0064] Soneson, C. , M. I. Love , and M. D. Robinson . 2016. “Differential Analyses for RNA‐Seq: Transcript‐Level Estimates Improve Gene‐Level Inferences.” F1000Research 4: 1521. 10.12688/f1000research.7563.2. PMC4712774 26925227

[ece372874-bib-0065] Spletter, M. L. , C. Barz , A. Yeroslaviz , et al. 2015. “The RNA‐Binding Protein Arrest (Bruno) Regulates Alternative Splicing to Enable Myofibril Maturation in Drosophila Flight Muscle.” EMBO Reports 16: 178–191. 10.15252/embr.201439791. 25532219 PMC4328745

[ece372874-bib-0066] Sprengelmeyer, Q. 2021. “The Population History of *Drosophila melanogaster* and the Evolution of Ethanol Tolerance and Body Size.” PhD Thesis, University of Wisconsin‐Madison.

[ece372874-bib-0067] Sprengelmeyer, Q. D. , S. Mansourian , J. D. Lange , et al. 2019. “Recurrent Collection of *Drosophila melanogaster* From Wild African Environments and Genomic Insights Into Species History.” Molecular Biology and Evolution 37: 627–638. 10.1093/molbev/msz271. PMC7038662 31730190

[ece372874-bib-0068] Sprengelmeyer, Q. D. , and J. E. Pool . 2021. “Ethanol Resistance in *Drosophila melanogaster* Has Increased in Parallel Cold‐Adapted Populations and Shows a Variable Genetic Architecture Within and Between Populations.” Ecology and Evolution 11: 15364–15376. 10.1002/ece3.8228. 34765183 PMC8571616

[ece372874-bib-0069] Sun, S.‐J. , A. M. Catherall , S. Pascoal , B. J. M. Jarrett , S. E. Miller , and M. J. Sheehan . 2019. “Ultra‐Local Adaptation due to Genetic Accommodation.” bioRxiv: 598292. 10.1101/598292.

[ece372874-bib-0070] Talikoti, A. 2021. “Identifying Genes Downstream of Mef2 That Influence Ethanol Sedation.” PhD Thesis, Virginia Commonwealth University.

[ece372874-bib-0071] Verta, J.‐P. , and A. Jacobs . 2022. “The Role of Alternative Splicing in Adaptation and Evolution.” Trends in Ecology & Evolution 37: 299–308. 10.1016/j.tree.2021.11.010. 34920907

[ece372874-bib-0072] Via, S. , and R. Lande . 1985. “The Evolution of Phenotypic Genotype‐Envrionment Interaction and Plasticity.” Evolution 39: 505–522. 10.1111/j.1558-5646.1985.tb00391.x. 28561964

[ece372874-bib-0073] West‐Eberhard, M. J. 2005. “Developmental Plasticity and the Origin of Species Differences.” Proceedings of the National Academy of Sciences 9: 6543–6549. 10.1073/pnas.0501844102. PMC1131862 15851679

